# Interleukin-4 Improves Metabolic Abnormalities in Leptin-Deficient and High-Fat Diet Mice

**DOI:** 10.3390/ijms21124451

**Published:** 2020-06-23

**Authors:** Shih-Yi Lin, Ching-Ping Yang, Ya-Yu Wang, Chiao-Wan Hsiao, Wen-Ying Chen, Su-Lan Liao, Yu-Li Lo, Yih-Hsin Chang, Chen-Jee Hong, Chun-Jung Chen

**Affiliations:** 1 Center for Geriatrics and Gerontology, Taichung Veterans General Hospital, Taichung City 407, Taiwan; sylin@vghtc.gov.tw; 2 Institute of Clinical Medicine, National Yang-Ming University, Taipei City 112, Taiwan; yywang@vghtc.gov.tw; 3 Department of Medical Research, Taichung Veterans General Hospital, Taichung City 407, Taiwan; milkygp@gmail.com (C.-P.Y.); slliao@vghtc.gov.tw (S.-L.L.); 4 Department of Family Medicine, Taichung Veterans General Hospital, Taichung City 407, Taiwan; 5 Department of Biotechnology and Laboratory Science in Medicine, National Yang-Ming University, Taipei City 112, Taiwan; hcw_alicia_1990@hotmail.com (C.-W.H.); cyh@ym.edu.tw (Y.-H.C.); 6 Department of Veterinary Medicine, National Chung-Hsing University, Taichung City 402, Taiwan; wychen@dragon.nchu.edu.tw; 7 Department and Institute of Pharmacology, National Yang-Ming University, Taipei City 112, Taiwan; yulilo@ym.edu.tw; 8 Institute of Brain Science, National Yang-Ming University, Taipei City 112, Taiwan; cjhong007@gmail.com; 9 Department of Medical Laboratory Science and Biotechnology, China Medical University, Taichung City 404, Taiwan; 10 Ph.D. Program in Translational Medicine, College of Life Sciences, National Chung Hsing University, Taichung City 402, Taiwan

**Keywords:** adiposity, inflammation, IL-4, leptin, obesity

## Abstract

Obesity is a metabolic disorder that results from complex interactions between genetic predisposition and dietary factors. Interleukin-4 (IL-4), besides its role in immunity, has metabolic effects on insulin efficacy. We studied the effects of IL-4 on metabolic abnormalities in a mice model of obesity involving leptin deficiency and leptin resistance. Leptin-deficient 145E and leptin-resistant high-fat diet (HFD) mice showed lower levels of circulating IL-4. 145E and HFD mice showed a number of abnormalities: Obesity, hyperglycemia, hyperinsulinemia, insulin resistance, dyslipidemia, liver injury, and adiposity with concurrent inflammation, decreases in Akt, signal transducer and activator of transcription 3 (STAT3), and STAT6 phosphorylation in the hypothalamus, liver, and epididymal fat. Independent of leptin-deficient obesity and dietary obesity, a course of 8-week IL-4 supplementation improved obesity and impairment in Akt, STAT3, and STAT6 signaling. Amelioration of cytokine expression, despite variable extents, was closely linked with the actions of IL-4. Additionally, the browning of white adipocytes by IL-4 was found in epididymal white adipose tissues and 3T3-L1 preadipocytes. Chronic exercise, weight management, and probiotics are recommended to overweight patients and IL-4 signaling is associated with clinical improvement. Thus, IL-4 could be a metabolic regulator and antiobesity candidate for the treatment of obesity and its complications.

## 1. Introduction

Obesity is a public health problem worldwide. Obesity is a risk factor that increases the prevalence and severity of several chronic disorders, predisposing subjects to a number of disorders, like cardiovascular diseases, hypertension, stroke, mood disorders, cancers, and type 2 diabetes [1,2,3]. With body weight management, obese subjects show marked improvements in obesity-related complications, in both human and rodent studies [4,5,6,7]. These phenomena underscore the importance of identifying strategies to avoid overweight, with the aim of combating obesity and its complications.

Energy balance is maintained through counter-regulations between food intake and energy expenditure via the communications of nutrients and hormones with distinct neuronal subpopulations and targeted tissues in the central nervous system (CNS) and the periphery. In the CNS, the critical neuronal populations are the anorexigenic proopiomelanocortin (POMC)- and orexigenic agouti-related protein (AgRP)-/neuropeptide Y (NPY)-expressing neurons, located in the arcuate nucleus of the hypothalamus. Regarding hormones, gut-derived ghrelin activates AgRP and NYP neurons, and inhibits the POMC neurons. In contrast, adipocyte-originating leptin has opposite effects [8,9,10]. When food nutrients exceed physiological requirements, the metabolized lipids are deposed at the intra-abdominal adipose tissues, particularly the white adipose tissue (WAT). The consequent adipocytic hyperplasia and hypertrophy increase the release of free fatty acids, proinflammatory cytokines, and adipokines into the blood stream, and lead to chronic and low-grade inflammations. The result is the impairment of insulin actions and glucose utility in the liver, skeletal muscles, and adipose tissues with a series of metabolic abnormalities, and the eventual development of obesity-associated complications [11,12,13,14,15,16,17]. Several studies have highlighted the crucial role of adipose tissues in metabolic changes, and their targeting for the prevention of obesity-induced complications.

Chronic exercise, weight management, and probiotics have all been implicated in improving obesity-related metabolic abnormalities. Interleukin-4 (IL-4) signaling is known to be closely linked to such beneficial effects [18,19,20,21]. Th2 cytokine IL-4 directs macrophages towards an anti-inflammatory phenotype and inhibits proinflammatory cytokine expression through receptor engagement and subsequent activation of the Janus kinase (Jak)/signal transducer and activator of transcription 6 (STAT6) axis [22]. In addition, deficiency of the insulin receptor substrate (IRS) impairs IL-4-induced M2 macrophage polarization, indicating a possible crosstalk between IL-4 and the IRS/PI3K/Akt axis [23]. An association has been described between the *IL-4/IL-4R* genotype and type 2 diabetes as well as between *IL-4* genotypes and high-density lipoprotein cholesterol [24,25]. IL-4 boosts insulin action in hepatocytes in vitro, and promotes myogenesis in myoblasts, resulting in greater insulin efficacy [26,27]. For adipocytes, IL-4 inhibits adipogenesis, promotes lipolysis, and restores insulin sensitivity against lipid-provoked insulin resistance [28,29,30]. Disruption of STAT6 impairs insulin actions in mice, and IL-4 administered intraperitoneally improves glucose and lipid metabolism in streptozotocin and high-fat diet (HFD) mice [15,31]. The above findings suggest the potential of IL-4 in combating obesity and its complications.

Leptin is a key hormone in regulating food intake and body weight homeostasis through the leptin/STAT3 signaling pathways [32]. Congenital leptin deficiency causes hyperphagia, early severe obesity, and metabolic disorder [33], and on the other hand, hyperleptinemia and leptin resistance can also be associated with common obesity [32]. In experimental studies, leptin-deficient ob/ob mice, leptin receptor-deficient db/db mice, and HFD-induced obese mice are common models used for the study of obesity [34]. Besides, rats harboring a deletion of the 14th amino acid Ile, and mice carrying a substitution of the 145th amino acid from Val to Glu in the leptin protein also show signs of obesity, hyperglycemia, hyperinsulinemia, and insulin resistance [35,36,37]. Evidence further indicates a promoting effect of leptin on IL-4 secretion [38].

In lean mice, the insulin-sensitive state of adipose tissue is proposed to preserve local IL-4 secretion by eosinophils and maintenance of an anti-inflammatory milieu. Whereas, adipose eosinophils and IL-4 expression were reduced in mice fed HFD or in mice with genetic obesity secondary to leptin deficiency, and there was an inverse relationship between adipose eosinophil numbers and mouse weight [39]. Particularly, it has been reported that adipose tissue macrophages from obese mice can exhibit an increased expression of the IL-4 receptor, and thus sensitivity to IL-4 [40]. Currently, it remains unclear whether IL-4 has beneficial effects against leptin deficiency- and leptin resistance-provoked metabolic abnormality and obesity. To extend the knowledge of biological implications of IL-4 on obesity and its complications, we studied the metabolic effects of IL-4 in two mice models of obesity: Leptin deficiency and HFD.

## 2. Results

### 2.1. Obese Mice Had a Lower Production of IL-4

**Figure 1:**
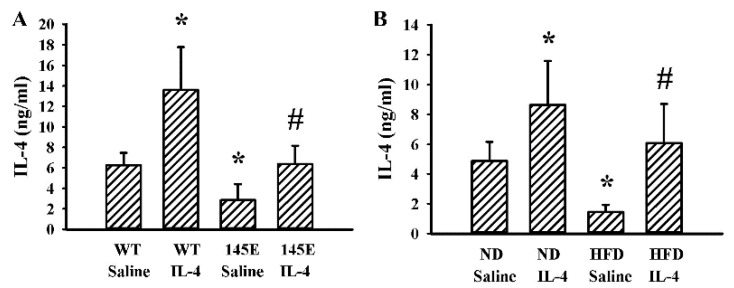
Obese mice showed reduced IL-4 expressions. (**A**) Wild-type C57BL/6 (WT) and leptin-deficient 145E mice were fed the normal diet (ND) for 8 weeks. Simultaneously, normal saline and IL-4 (1 μg/mouse) were intraperitoneally administrated twice a week. Blood samples were collected and subjected to ELISA for measuring IL-4 levels. * *p* < 0.05 vs. WT/saline group and # *p* < 0.05 vs. 145E/saline group, *n* = 6. (**B**) C57BL/6 mice were fed with the normal diet (ND) or high-fat diet (HFD) for 12 weeks. Simultaneously, normal saline and IL-4 (1 μg/mouse) were intraperitoneally administrated twice a week for the last 8 weeks. Blood samples were collected and subjected to ELISA for the measurement of IL-4 levels. * *p* < 0.05 vs. ND/saline group and # *p* < 0.05 vs. HFD/saline group, *n* = 6.

To initiate a study surrounding IL-4 effects on metabolic changes, we therefore determined the endogenous levels of IL-4 in both lean and obese mice. Obese mice, both 145E (Figure 1A) and HFD (Figure 1B), had lower circulating levels of IL-4 than lean mice. Independent of being lean or obese, exogenous IL-4 supplementation increased the circulating level in the bloodstream (Figure 1A,B). Therefore, obese mice had a lower level of endogenous IL-4 in their blood circulation.

### 2.2. IL-4 Ameliorated Metabolic Alterations in Leptin-Deficient Mice

**Figure 2:**
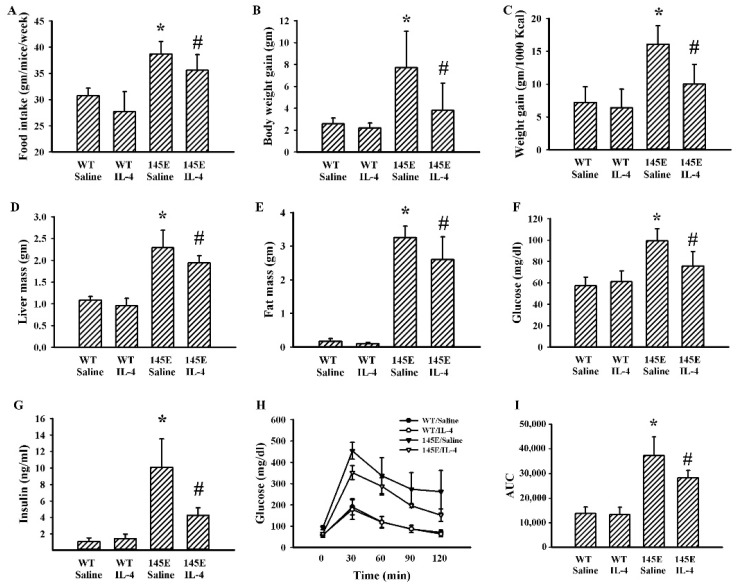
IL-4 ameliorated metabolic changes in the leptin-deficient mice. Wild type C57BL/6 (WT) and leptin-deficient 145E mice were fed the normal diet (ND) for 8 weeks. Simultaneously, normal saline and IL-4 (1 μg/mouse) were intraperitoneally administrated twice a week. The average food intake (**A**), body weight gain (**B**), feeding efficacy (**C**), liver mass (**D**), and epididymal fat mass (**E**) were measured. Blood samples were collected from 8-h fasting mice and fasting glucose (**F**) and insulin (**G**) levels measured. The 8-h fasting mice were intraperitoneally injected with a glucose solution (2 g/kg). Blood samples were collected from the tail veins at the indicated times after treatments and the levels of glucose were measured (**H**). AUC of the glucose–time curves was calculated (**I**). * *p* < 0.05 vs. WT/saline group and # *p* < 0.05 vs. 145E/saline group, *n* = 6.

Within 8 weeks of feeding, 145E mice showed increases in food intake (Figure 2A), body weight (Figure 2B), feeding efficiency (Figure 2C), liver mass (Figure 2D), epididymal fat mass (Figure 2E), fasting glucose (Figure 2F), and insulin (Figure 2G), as well as impaired glucose tolerance (Figure 2H,I). IL-4 had little effect on WT lean mice, while it ameliorated the aforementioned metabolic alterations in 145E obese mice (Figure 2). That is, IL-4 displays improvement effects against metabolic alterations in leptin-deficient 145E obese mice.

### 2.3. IL-4 Reduced Hypothalamic Changes in Leptin-Deficient Mice

**Figure 3:**
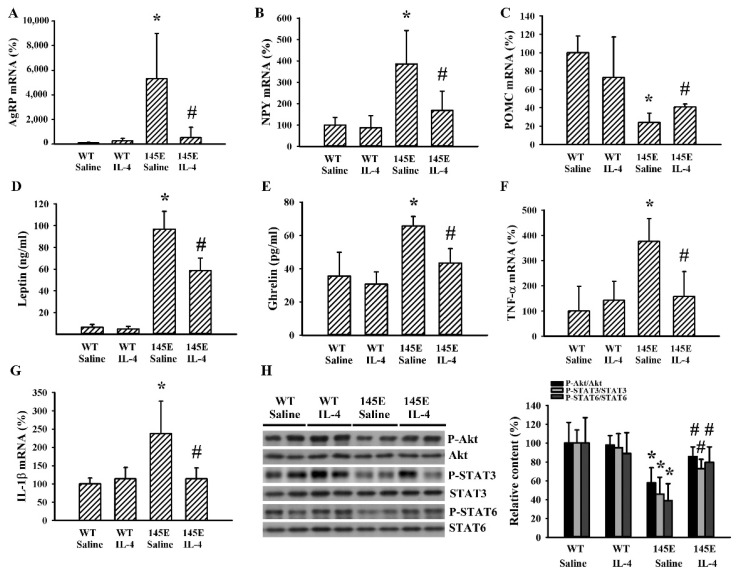
IL-4 ameliorated hypothalamic changes in leptin-deficient mice. Wild-type C57BL/6 (WT) and leptin-deficient 145E mice were fed the normal diet (ND) for 8 weeks. Simultaneously, normal saline and IL-4 (1 μg/mouse) were intraperitoneally administrated twice a week. Total RNAs were extracted from the hypothalamic tissues and subjected to qRT-PCR for the measurement of AgRP (**A**), NPY (**B**), POMC (**C**), TNF-α (**F**), and IL-1β (**G**) mRNA expression. Blood samples were collected and subjected to ELISA for the measurement of leptin (**D**) and ghrelin (**E**) levels. Proteins were extracted from the hypothalamic tissues and subjected to Western blot analysis with the indicated antibodies. Representative blots and quantitative data are shown (**H**). * *p* < 0.05 vs. WT/saline group and # *p* < 0.05 vs. 145E/saline group, *n* = 6.

In the hypothalamus of 145E mice, we found increased levels in the mRNA of orexigenic AgRP (Figure 3A) and NPY (Figure 3B), and decreased levels in the mRNA of anorexigenic POMC (Figure 3C). These observed changes were reduced by IL-4. Parallel increases in 145E mice and decreases by IL-4 were found in the circulating levels of leptin (Figure 3D) and ghrelin (Figure 3E) as well as mRNA levels of tumor necrosis factor-α (TNF-α) (Figure 3F) and interleukin-1β (IL-1β) (Figure 3G) in hypothalamic tissues. In addition, levels of protein phosphorylation in Akt, STAT3, and STAT6 decreased in the hypothalamic tissues of 145E mice but were reversed by IL-4 (Figure 3H). Findings indicated that the promotion of orexigenic and reduction of anorexigenic signaling in the hypothalamus of 145E mice were accompanied by increased mRNA expression of TNF-α/IL-1β cytokines and impaired phosphorylation of Akt, STAT3, and STAT6. These changes were ameliorated by IL-4.

### 2.4. IL-4 Reduced Hepatic Changes in Leptin-Deficient Mice

**Figure 4:**
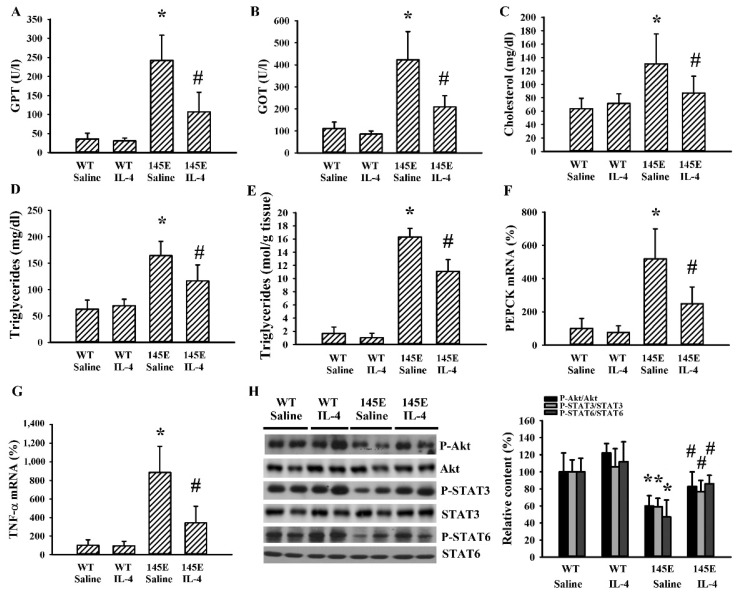
IL-4 ameliorated hepatic changes in leptin-deficient mice. Wild-type C57BL/6 (WT) and leptin-deficient 145E mice were fed the normal diet (ND) for 8 weeks. Simultaneously, normal saline and IL-4 (1 μg/mouse) were intraperitoneally administrated twice a week. Blood samples were collected and GPT (**A**), GOT (**B**), total cholesterol (**C**), and triglycerides (**D**) measured. Hepatic tissues were collected and subjected to measurement of triglycerides (**E**). Total RNAs were extracted from the hepatic tissues and subjected to qRT-PCR for the determination of PEPCK (**F**) and TNF-α (**G**) mRNA expressions. Proteins were extracted from the hepatic tissues and subjected to Western blot analysis with indicated antibodies. Representative blots and quantitative data are shown (**H**). * *p* < 0.05 vs. WT/saline group and # *p* < 0.05 vs. 145E/saline group, *n* = 6.

Changes of hepatic metabolism in obesity are signs of metabolic abnormalities [36]. Serum GPT (Figure 4A), GOT (Figure 4B), total cholesterol (Figure 4C), and triglyceride (Figure 4D) levels were found to be higher in 145E mice than in WT mice. Hepatic tissues of 145E mice displayed an elevated accumulation of triglycerides (Figure 4E), mRNA expressions of phosphoenolpyruvate carboxykinase (PEPCK) (Figure 4F) and TNF-α (Figure 4G), as well as reduced protein phosphorylation of Akt, STAT3, and STAT6 (Figure 4H). IL-4 ameliorated all these abnormal changes (Figure 4). The findings suggested a reversal effect of IL-4 on hepatic metabolic abnormalities and decreased Akt, STAT3, and STAT6 signaling in 145E obese mice.

### 2.5. IL-4 Reduced Adipocytic Changes in Leptin-Deficient Mice

**Figure 5:**
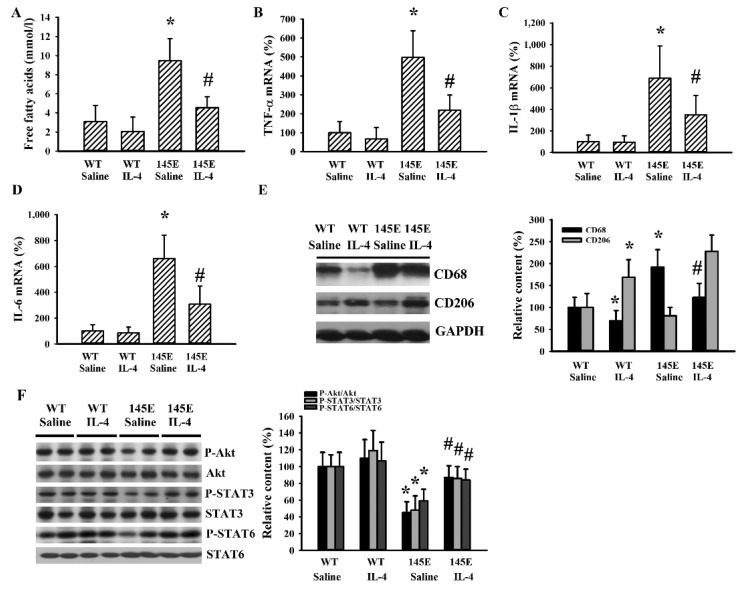
IL-4 ameliorated adipocytic changes in leptin-deficient mice. Wild-type C57BL/6 (WT) and leptin-deficient 145E mice were fed the normal diet (ND) for 8 weeks. Simultaneously, normal saline and IL-4 (1 μg/mouse) were intraperitoneally administrated twice a week. Blood samples were collected and free fatty acids (**A**) measured. Total RNAs were extracted from the epididymal fat tissues and mRNA expressions measured with qRT-PCR for TNF-α (**B**), IL-1β (**C**), and IL-6 (**D**). Proteins were extracted from the epididymal fat tissues and subjected to Western blot analysis with indicated antibodies. Representative blots and quantitative data are shown (**E**,**F**). * *p* < 0.05 vs. WT/saline group and # *p* < 0.05 vs. 145E/saline group, *n* = 6.

Adipose tissues are sites for lipid deposition and metabolism [29,30]. 145E obese mice were found to have higher circulating levels of free fatty acids and these increments were reduced by IL-4 (Figure 5A). In the epididymal fats of 145E mice, mRNA levels were elevated with TNF-α (Figure 5B), IL-1β (Figure 5C), and IL-6 (Figure 5D); the protein level was increased with cluster of differentiation 68 (CD68) (Figure 5E), while protein phosphorylation was lowered with Akt, STAT3, and STAT6 (Figure 5E). All these abnormal changes in mRNA, protein level, and protein phosphorylation were reduced by IL-4 (Figure 5B–F). Intriguingly, the protein level of CD206 was increased in the presence of IL-4 (Figure 5E). The results are consistent with the higher secretion of free fatty acid and higher cytokine mRNA expression, along with impaired Akt, STAT3, and STAT6 signaling in epididymal fats of 145E mice. These changes were reduced by IL-4.

### 2.6. IL-4 Reduced Metabolic Changes in the HFD Mice

**Figure 6:**
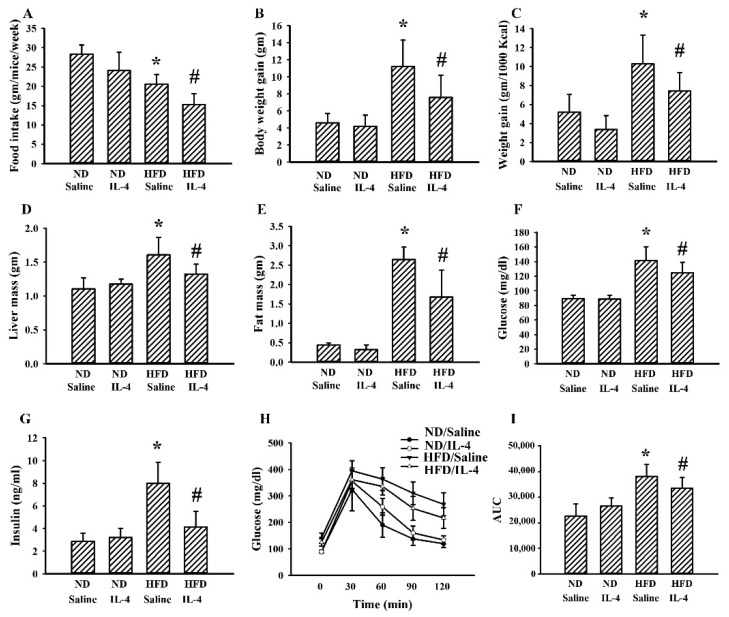
IL-4 ameliorated metabolic changes in leptin-resistant mice. C57BL/6 mice were fed the normal diet (ND) or high-fat diet (HFD) for 12 weeks. Simultaneously, normal saline and IL-4 (1 μg/mouse) were intraperitoneally administrated twice a week for the last 8 weeks. The average food intake (**A**), body weight gain (**B**), feeding efficacy (**C**), liver mass (**D**), and epididymal fat mass (**E**) were measured. Blood samples were collected from 8-h fasting mice and fasting glucose (**F**) and insulin (**G**) levels measured. The 8-h fasting mice were intraperitoneally injected with a glucose solution (2 g/kg). Blood samples were collected from the tail veins at the indicated times after treatments and levels of glucose measured (**H**). AUC of the glucose–time curves was calculated (**I**). * *p* < 0.05 vs. ND/saline group and # *p* < 0.05 vs. HFD/saline group, *n* = 6.

**Figure 7:**
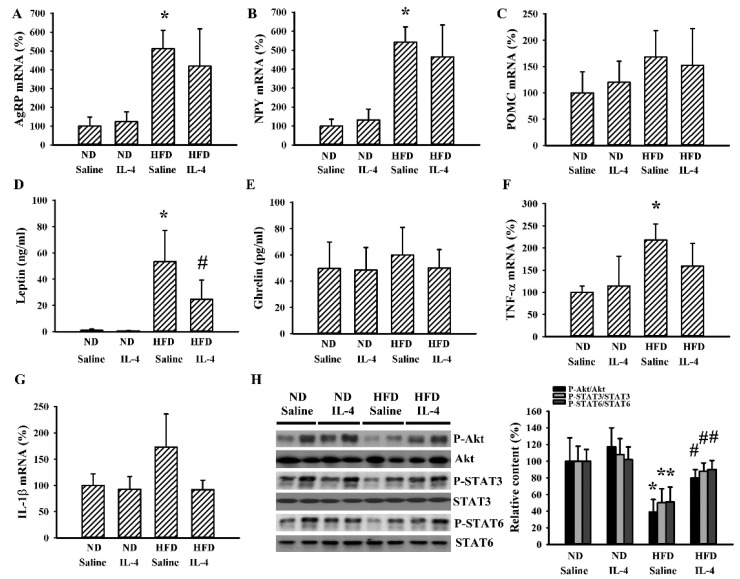
IL-4 ameliorated hypothalamic changes in leptin-resistant mice. C57BL/6 mice were fed the normal diet (ND) or high-fat diet (HFD) for 12 weeks. Simultaneously, normal saline and IL-4 (1 μg/mouse) were intraperitoneally administrated twice a week for the last 8 weeks. Total RNA were extracted from the hypothalamic tissues and mRNA expressions measured with qRT-PCR for AgRP (**A**), NPY (**B**), POMC (**C**), TNF-α (**F**), and IL-1β (**G**). Blood samples were collected and leptin (**D**) and ghrelin (**E**) levels measured with ELISA. Proteins were extracted from the hypothalamic tissues and subjected to Western blot analysis with indicated antibodies. Representative blots and quantitative data are shown (**H**). * *p* < 0.05 vs. ND/saline group and # *p* < 0.05 vs. HFD/saline group, *n* = 6.

**Figure 8:**
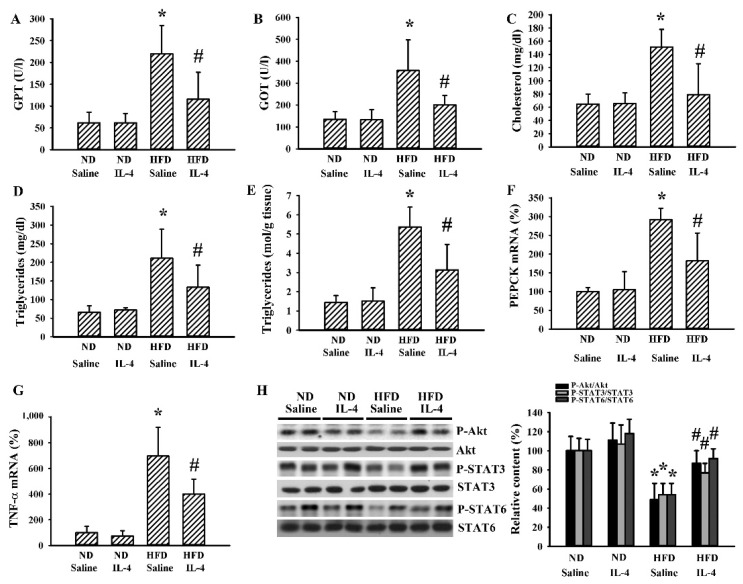
IL-4 ameliorated hepatic changes in leptin-resistant mice. C57BL/6 mice were fed the normal diet (ND) or high-fat diet (HFD) for 12 weeks. Simultaneously, normal saline and IL-4 (1 μg/mouse) were intraperitoneally administrated twice a week for the last 8 weeks. Blood samples were collected and GPT (**A**), GOT (**B**), total cholesterol (**C**), and triglyceride (**D**) levels measured. Hepatic tissues were collected and subjected to measurement of triglycerides (**E**). Total RNAs were extracted from the hepatic tissues and mRNA expression measured with qRT-PCR for PEPCK (**F**) and TNF-α (**G**). Proteins were extracted from the hepatic tissues and subjected to Western blot analysis with indicated antibodies. Representative blots and quantitative data are shown (**H**). * *p* < 0.05 vs. ND/saline group and # *p* < 0.05 vs. HFD/saline group, *n* = 6.

**Figure 9:**
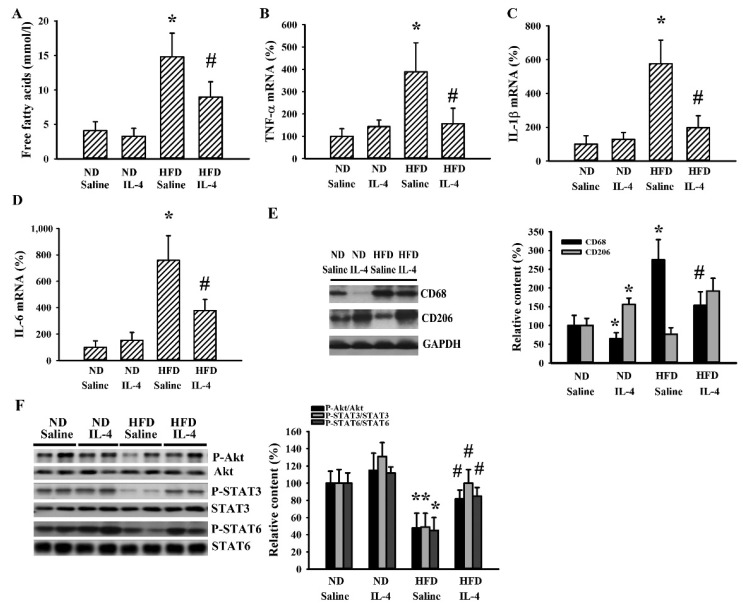
IL-4 ameliorated adipocytic changes in leptin-resistant mice. C57BL/6 mice were fed the normal diet (ND) or high-fat diet (HFD) for 12 weeks. Simultaneously, normal saline and IL-4 (1 μg/mouse) were intraperitoneally administrated twice a week for the last 8 weeks. Blood samples were collected and subjected to the measurement of free fatty acids (**A**). Total RNA were extracted from the epididymal fat tissues and mRNA expression measured with qRT-PCR for TNF-α (**B**), IL-1β (**C**), and IL-6 (**D**). Proteins were extracted from the epididymal fat tissues and subjected to Western blot analysis with indicated antibodies. Representative blots and quantitative data are shown (**E**,**F**). * *p* < 0.05 vs. ND/saline group and # *p* < 0.05 vs. HFD/saline group, *n* = 6.

Although 145E mice release leptin into the blood circulation, the mutant leptin loses its biological activity [36]. To explore whether IL-4′s effects are common to obese populations, HFD obese mice were analyzed for comparison. In these mice, IL-4 showed similar effects regarding food intake (Figure 6A), body weight gain (Figure 6B), feeding efficiency (Figure 6C), liver mass (Figure 6D), epididymal fat mass (Figure 6E), fasting glucose (Figure 6F), insulin (Figure 6G), and glucose tolerance (Figure 6H and 6I). In the case of HFD obese mice, when compared with the ND lean mice, they showed elevated mRNA expressions of AgRP (Figure 7A) and NPY (Figure 7B) in the hypothalamus while mRNA expression of POMC remained unchanged. In these HFD mice, we only found an increased level of circulating leptin (Figure 7D) but not ghrelin (Figure 7E). Additionally, in their hypothalamic tissues, we found a moderate elevation in the expression of TNF-α mRNA (Figure 7F) but unchanged expression of IL-1β mRNA (Figure 7G). Compared with ND mice, these HFD mice had decreased protein phosphorylation of Akt, STAT3, and STAT6 (Figure 7H) in the hypothalamic tissues. Intriguingly, IL-4 displayed ameliorative effects only on leptin (Figure 7D) and protein phosphorylation (Figure 7H). Regarding the changes in the hepatic (Figure 8) and adipocytic (Figure 9) tissues, IL-4 and HFD mice displayed the same effects as those in IL-4 and leptin-deficient mice. The findings are consistent with the HFD and 145E mice sharing most metabolic alterations, except some parts of the hypothalamic signaling pathways.

### 2.7. IL-4 Promoted White Adipocyte Browning

**Figure 10:**
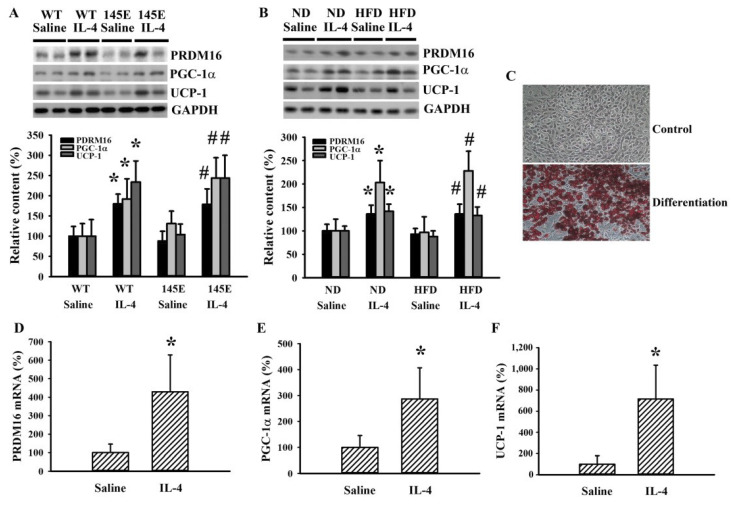
IL-4 promoted white adipocyte browning. (**A**) Wild-type C57BL/6 (WT) and leptin-deficient 145E mice were fed the normal diet (ND) for 8 weeks. Simultaneously, normal saline and IL-4 (1 μg/mouse) were intraperitoneally administrated twice a week. Proteins were extracted from the epididymal fat tissues and subjected to Western blot analysis with indicated antibodies. * *p* < 0.05 vs. WT/saline group, and # *p* < 0.05 vs. 145E/saline group, *n* = 6. (**B**) C57BL/6 mice were fed the normal diet (ND) or high-fat diet (HFD) for 12 weeks. Simultaneously, normal saline and IL-4 (1 μg/mouse) were intraperitoneally administrated twice a week for the last 8 weeks. Proteins were extracted from the epididymal fat tissues and subjected to Western blot analysis with indicated antibodies. * *p* < 0.05 vs. ND/saline group and # *p* < 0.05 vs. HFD/saline group, *n* = 6. (**C**) Postconfluent 3T3-L1 cells differentiated into mature adipocytes, as described in the methods. The undifferentiated control and differentiated cells were stained with Oil Red O. Postconfluent 3T3-L1 cells were differentiated into mature adipocytes in the presence of IL-4 (0 and 10 ng/mL), as described in the methods. Total RNA were extracted and mRNA expression measured with qRT-PCR for PRDM16 (**D**), PGC-1α (**E**), and UCP-1 (**F**). * *p* < 0.05 vs. saline group, *n* = 4.

White adipose tissue is a storage site of surplus energy, and brown adipose tissue consumes energy to generate heat. The conversion of white adipose to brown adipose tissues is inversely correlated with adiposity and metabolic abnormality [11,16,17]. To explore the effects of IL-4 on metabolic regulation, we determined the expression of molecules regulating white adipocyte browning in epididymal fats. IL-4 caused elevated levels of PR domain containing 16 (PRDM16), peroxisome proliferator-activated receptor gamma coactivator 1α (PGC-1α), and uncoupling protein 1 (UCP-1) proteins compared with controls in both 145E (Figure 10A) and HFD (Figure 10B) mice. In the in vitro cell model, 3T3-L1 preadipocytes differentiated into mature adipocytes (Figure 10C). During differentiation, IL-4 increased mRNA expression of PRDM16 (Figure 10D), PGC-1α (Figure 10E), and UCP-1 (Figure 10F). The findings are consistent with the promoting effect of IL-4 on white adipocyte browning.

## 3. Discussion

Leptin is a pivotal hormone regulating food intake and body weight by coordinating communications between the hypothalamus and the periphery. Leptin deficiency is a result of a genetic mutation predisposing subjects to severe obesity, while leptin resistance is linked to dietary obesity [32,33,34,35,36,37,38,41]. In this study, we found that obese 145E and HFD mice had lower circulating levels of IL-4 than wild-type and lean mice. A course of 8-week IL-4 supplementation improves common phenotypes of obesity and impaired signaling pathways of Akt, STAT3, and STAT6 in the hypothalamic, hepatic, and epididymal fat tissues. Despite the inconsistent results across studies, amelioration of cytokine expression and promotion of white adipocyte browning have been shown to have a close link with IL-4. Since obesity is associated with lower circulating levels of IL-4, our current findings further highlighted IL-4 being an alternative candidate in combating obesity and its complications.

Consistent with a previous report [36], both NPY and AgRP mRNA levels in the hypothalamus were significantly increased in 145E mice, while POMC mRNA tended to be lower in 145E mice than in wild-type mice. Besides, the changes in hypothalamic orexigenic/anorexigenic peptides and cytokines along with circulating ghrelin in the leptin-deficient 145E mice were more marked than in HFD mice. It has been reported that exposure to HFD-induced obesity can cause dysregulation of the expression of NPY and AgRP in the hypothalamus [42,43]. However, the hypothalamic anorexigenic POMC can also be induced as an adaptive mechanism against obesity [44,45]. Therefore, in the HFD obese animals, although later eating less, they still consumed more calories, and gained more weight and adipose tissue mass.

Leptin is primarily secreted by white adipose tissues and its circulating level is proportional to body fat mass. The interactions of leptin with its longest receptor isoform trigger STAT3 signaling in the hypothalamus, liver, and adipose tissues to coordinate food intake, energy expenditure, and lipid or glucose metabolism. Its actions are compromised by defective mutation, receptor mutation, resistance, and inflammation [9,14,35,36,41]. We previously identified and created a model with leptin mutation, the 145E mice, by replacing Val by Glu in the codon 145. These mice showed metabolic abnormalities and developed obese phenotypes. Intraperitoneal injection with wild-type leptin reduced food intake [36]. Here, we further demonstrated in 145E mice that hyperphagia, hyperglycemia, hyperinsulinemia, insulin resistance, dyslipidemia, hepatic steatosis, and adiposity were all accompanied by elevated cytokine expressions and lowered phosphorylation of Akt, STAT3, and STAT6 in the hypothalamus, liver, and epididymal fat. Such changes in the tissues towards proinflammation and impairment of Akt, STAT3, and STAT6 signaling were all duplicated in dietary obesity. As leptin can mediate the immune response [46], it was speculated that proinflammatory stress in various tissues of 145E mice might be related to their leptin deficiency status. Consistent with an earlier report [47], in HFD obese mice, we found a lesser trend of increased expression of cytokine mRNA in the hypothalamus than in peripheral organs, although several studies in HFD rodent models have indicated not only peripheral inflammation but also increased inflammatory signaling in the hypothalamus, which was linked to the development of central leptin resistance and obesity [48]. Taken altogether, in the current study, it was shown that leptin deficiency and leptin resistance shared some but not all metabolic changes in the development of obesity.

IL-4 is a Th2 cytokine that can promote M2 macrophage polarization in adipose tissues [12,23], and it has been proposed that IL-4 signaling could be a potential target to sustain insulin sensitivity in obesity [40]. A large amount of literature documents the effect of IL-4 signaling to block immune-mediated inflammation in both peripheral tissues and the brain [49,50]. In leptin-deficient obese mice, the expression of hypothalamic orexigenic/anorexigenic peptides and inflammatory cytokines as well as signaling pathways of Akt, STAT3, and STAT6 in the hypothalamus, body weight, and glucose dysregulation after IL-4 administration was restored towards those in wild-type mice along with circulating ghrelin. On the contrary, there seemed to be less effects of IL-4 on hypothalamic orexigenic/anorexigenic peptides and inflammatory cytokines expression, except preserved higher phosphorylation levels of Akt, STAT3, and STAT6 in the hypothalamus in HFD obese mice, although their weight was also lowered. In fact, a previous study reported that central administration of IL-4 exacerbated hypothalamic inflammation and weight gain during high-fat feeding [47]. These findings indicated the central sensitivity of IL-4 in leptin-deficient and -resistant obese animals might be different. The above issues warrant further investigation.

It was speculated that body weight loss as well as restoration of metabolic phenotypes in leptin-deficient and HFD obese mice after IL-4 administration might come from other effects on peripheral organs (e.g., liver and adipose tissues, and pancreatic islets). Disruption of STAT6, downstream of IL-4, inhibits insulin action in mice, and IL-4 enhances insulin efficacy on adipocytes, hepatocytes, myoblasts, and diabetic mice [15,26,27,28,29,30,31]. Akt, a crucial member of insulin action, is also a candidate target of IL-4 signaling [22,23]. Exogenous IL-4 preserved higher phosphorylation levels of Akt, STAT3, and STAT6 in the liver and epididymal fat of both 145E and HFD obese mice. Since typical leptin/STAT3 and IL-4/STAT6 are closely linked to insulin/Akt in metabolic regulation, our present findings are consistent with IL-4 being able to affect the STAT3 and STAT6 cascades and Akt under certain situations while it is ineffective in WT and lean mice. In addition, IL-4 protects pancreatic beta cells from injury and the Th2 cytokine IL-13 from interfering in gluconeogenic enzyme expression via STAT3 [51,52]. In 145E and HFD mice, elevation in the mRNA levels of the hepatic gluconeogenic enzyme PEPCK was reduced by IL-4. On top of the improved insulin/Akt action, the direct effect of IL-4/STAT3 on the gluconeogenic enzyme might also lower the glucose output.

White adipose tissues are central to metabolic regulation through the release of adipokines. It is known that obesity is associated with chronic low-grade inflammation of the adipose tissue and increased M1 macrophages. However, a recent paper reported that locally proliferating macrophages in obese adipose were not classically activated (M1, CD68 positive), but rather they exhibited an alternatively activated (M2, CD206 positive) immune phenotype with increased gene expression of the IL-4 receptor [40]. Obese adipose tissues showed an increased CD68 content, while it had minor effect on the CD206 content. IL-4 not only decreased the increment of CD68 but also elevated CD206, indicating the suppression of M1 macrophages and promotion of M2 macrophages. Based on this, adipose sensitivity to IL-4 stimulation can be enhanced by increased phosphorylation of STAT6, lipolysis in fat cells, and insulin sensitivity [31]. Currently, white adipocyte browning is an emerging strategy to improve metabolic abnormalities. PDM16 is a zinc-finger nuclear protein that controls transcriptional programs at the differentiating brown adipocytes and mitochondrial biogenesis involving PGC-1α and UCP-1. The processes of adipocyte browning are accompanied by STAT6 hyperphosphorylation [16]. The leptin/STAT3 signaling pathways coordinate and promote the browning of white adipocytes [11,17]. In the epididymal white adipose tissues and 3T3-L1 cells, IL-4 elevated the mRNA expression and protein levels of PRDM16, PGC-1α, and UCP-1. As IL-4 increased STAT3 and STAT6 phosphorylation, adipocyte browning was consequently achieved.

In WT and lean mice, generally, there seemed little effects of IL-4 on weight, intake, glucose tolerance, leptin, ghrelin, and inflammatory cytokines as shown in obese mice. It was possible that chronic IL-4 administration in lean animals may elicit compensatory responses in the body with a final neutral metabolic influence. Besides, even in control animals, many factors, such as fasting and feeding duration, stress, and environment, can all transiently affect the expression of circulating leptin, ghrelin, glucose levels, and inflammatory cytokines, and thus a more careful standardization is necessary to avoid such variations [53]. Nevertheless, the regulatory effects of IL-4 on metabolism in animals with a non-obese status need further investigation.

In clinical practice, chronic exercise, weight management, and probiotics are recommended to overweight patients. IL-4 signaling is one parameter associated with clinical improvement [18,19,20,21]. Through this study, we provided experimental evidence showing that IL-4 induced improvements of metabolic abnormalities in a mice model of obesity with leptin deficiency and HFD. The models of 145E and HFD obese mice showed lower levels of circulating IL-4. The metabolic abnormalities in 145E and HFD obese mice were accompanied by inflammation and reduced Akt, STAT3, and STAT6 signaling in the hypothalamus, liver, and epididymal fat. IL-4-mediated improvements were found in parallel with the amelioration of inflammation and the promotion of Akt, STAT3, and STAT6 phosphorylation. Additionally, the browning of white adipocyte by IL-4 was found in epididymal white adipose tissues and 3T3-L1 preadipocytes. Despite some limitations of our experiments and some inconsistency with the literature, our results suggested that the Th2 cytokine IL-4 is a metabolic regulator and antiobesity candidate for the treatment of obesity and its complications. It should be noted that IL-4 can exert systemic effects around inflammation. Therefore, before IL-4 can be translated into clinical applications, a deeper investigative insight into its metabolic actions is still required.

## 4. Materials and Methods

### 4.1. Animals and Treatments

Adult male leptin-deficient Leptin^145E/145E^ (145E) mice [36] and species-matched wild-type (WT) C57BL/6 mice were housed in a controlled animal facility and handled according to procedures approved by the Animal Experimental Committee of Taichung Veterans General Hospital (IACUC No. La-1081629, 17 April, 2019). Mice of WT (*n* = 24) and 145E (*n* = 24) strains, aged 8 weeks old, were fed with a normal diet (ND, LabDiet 5001, 13% energy provided by fat) for 8 weeks. Simultaneously, normal saline and IL-4 (1 μg/mouse) were intraperitoneally administrated twice a week (see previously reported protocols for details [31]). For diet-induced obesity, C57BL/6 mice (8-week-old) were fed with ND (*n* = 24) or HFD (*n* = 24) (TestDiet, 58Y1, 61% energy provided by fat) for 12 weeks. Normal saline and IL-4 (1 μg/mouse) were intraperitoneally administrated twice a week during the last 8 weeks.

### 4.2. Cell Culture and Differentiation

The 3T3-L1 preadipocytes were maintained in Dulbecco′s modified eagle medium (DMEM) containing 10% calf serum. For adipogenesis, postconfluent cells (2 days old) were maintained in DMEM by adding 0.5 mM 3-isobutyl-1-methyxantine (IBMX) IBMX, 1 μM dexamethasone, 10 μg/mL insulin, and 10% fetal bovine serum (FBS) for 2 days. Thereafter, the cultured media was switched to DMEM containing 5 μg/mL insulin and 10% FBS and changed every 2 days. Cells were analyzed 8 days later. IL-4 (10 ng/mL) and vehicle were added to the cells during the 2-day induction period. Treatments and Oil Red O staining were performed in accordance with previously reported methods with slight modifications [30].

### 4.3. Glucose Tolerance Test

For the intraperitoneal glucose tolerance test (IPGTT) (*n* = 6/group), mice were fasted for 8 h and intraperitoneally given glucose solution (2 g/kg body weight). Blood samples were collected from the tail veins at specified time points, and glucose levels were determined using a hand-held Accucheck glucometer (Roche Diagnostics, Indianapolis, IN, USA). The total area under the curve (AUC) for IPGTT was calculated using the trapezoidal (trapezium) rule.

### 4.4. Blood Sample Analyses

Mice (*n* = 6/group) were anesthetized with isoflurane (2–4%), blood samples were withdrawn from the left femoral artery, and the serum was kept at −80 °C until analyses. Serum levels of insulin (Shibayagi, Gunma, Japan), leptin, ghrelin, and medium level of IL-4 (R&D Systems, Minneapolis, MN, USA) were measured using the Enzyme-Linked Immunosorbent Assay (ELISA) kit, according to the manufacturer′s instructions. Serum levels of total cholesterol (Cholesterol E, Wako, Osaka, Japan), free fatty acids (NEFA C, Wako, Osaka, Japan), triglycerides (Stanbio Laboratory, San Antonin, TX, USA), and GPT/GOT were determined using the Alanine Aminotransferase Activity Assay Kit (ALT/GPT)/Aspartate Aminotransferase Activity Assay Kit (AST/GOT), MyBioSource, San Diego, CA, USA.

### 4.5. RNA Isolation and Quantitative Real-Time Reverse Transcriptase-Polymerase Chain Reaction (qRT-PCR)

Hypothalami, epididymal fats, liver (*n* = 6/group), and 3T3-L1 cells were isolated and stored in RNAlater solution (Ambion, Austin, TX, USA). RNAs were extracted using the TRIzol Reagent (Invitrogen, Carlsbad, CA, USA). Levels of mRNA were analyzed with SYBR green-based qRT-PCR (Applied Biosystems, Foster City, CA, USA), and the internal control was *β-actin*. Primers used for amplifications were as follows: AgRP 5′-CGGAGGTGCTAGATCCACAGAA-3′ and 5′-AGGACTCGTGCAGCCTTACAC-3′; NPY 5′-AGAGATCCAGCCCTGAGACA-3′ and 5′-TTTCATTTCCCATCACCACA-3′; POMC 5′-GTTACGGTGGCTTCATGACCTC-3′ and 5′-CGCGTTCTTGATGATGGCGTTC-3′; TNF-α 5′-TCTTCTCATTCCTGCTTGTGG-3′ and 5′-GGTCTGGGCCATAGAACTGA-3′; IL-1β 5′-TTCATCTTTGAAGAAGAGCCCAT-3′ and 5′-TCGGAGCCTGTAGTGCAGTT-3′; PEPCK 5′-GAACACACCCTCGGTCAACA-3′ and 5′-CAAGGTCATCCAGGGCAGCCTC-3′; IL-6 5′-TGATGGATGCTACCAAACTGG-3′ and 5′-TTCATGTACTCCAGGTAGCTATGG-3′; PRDM16 5′-GATGGGAGATGCTGACGGAT-3′ and 5′-TGATCTGACACATGGCGAGG-3′, PGC-1α 5′-TCCTCTGACCCCAGACTCAC-3′ and 5′-TAGAGTCTTGGAGCTCCT-3′; UCP-1 5′-GAAAGGGACCCCTAATC-3′ and 5′-GGGACGTCATCTGCCAGTA-3′ and β-actin 5′-CCTCTATGCCAACACAGTGCTGTCT-3′ and 5′-GCTCAGGAGGAGCAATGATCTTGA-3′.

### 4.6. Western Blot Analysis

Hypothalami, epididymal fats, and liver tissues (*n* = 6/group) were isolated, and proteins extracted using the commercial Tissue Protein Extraction Reagents (T-PER, Pierce Biotechnology, Rockford, IL, USA). Equal amounts of proteins were resolved by SDS-PAGE, before being transferred to the polyvinylidine fluoride membrane. The membranes were blocked with 5% nonfat milk and incubated with primary antibodies, horseradish peroxidase-conjugated IgG, and enhanced chemiluminescence Western blotting reagents. The visualized signals were quantitated by a densitor meter. Primary antibodies used were: Akt, phospho-Akt, signal transducers and activators of transcription 3 (STAT3), phospho-STAT3, STAT6, phospho-STAT6, PRDM16, PGC-1α, UCP-1, CD68, CD206, and glyceraldehyde 3-phosphate dehydrogenase (GAPDH) (Santa Cruz Biotechnology, Santa Cruz, CA, USA).

### 4.7. Statistical Analyses

Statistical results were expressed as mean ± standard deviation. The one-way analysis of variance (ANOVA) was performed to compare inter-group differences. Then, Dunnett′s test or Tukey post-hoc test was performed for the purpose of comparison. Statistically significant differences were set at *p* < 0.05.

## Abbreviations

AgRP	Agouti-Related Protein
AUC	Area Under Curve
CNS	Central Nervous System
DMEM	Dulbecco′s Modified Eagle Medium
ELISA	Enzyme-Linked Immunosorbent Assay
GAPDH	Glyceraldehyde 3-Phosphate Dehydrogenase
HFD	High-Fat Diet
IGPTT	Intraperitoneal Glucose Tolerance Test
IL-1β	Interleukin-1β
IL-4	Interleukin-4
IRS	Insulin Receptor Substrate
Jak	Janus Kinase
NPY	Neuropeptide Y
PEPCK	Phosphoenolpyruvate Carboxykinase
PGC1α	Peroxisome Prolioferator-Activated Receptor Gamma Coactivator 1α
POMC	Pro-Opiomelanocortin
PRDM16	PR-Domain Containing 16
RT-PCR	Reverse Transcriptase-Polymerase Chain Reaction
STAT3	Signal Transducer and Activator of Transcription 3
TNF-α	Tumor Necrosis Factor-α
UCP-1	Uncoupling Protein 1
WAT	White Adipose Tissue
